# TMEM150C and F2R as transcriptomic signatures for refractoriness to 7 + 3 induction therapy in acute myeloid leukemia

**DOI:** 10.3389/fonc.2026.1926332

**Published:** 2026-10-23

**Authors:** Yu-Jeong Choi, Jiwoo Kim, Xuan Linh Mai, Arvie Camille V. de Guzman, Yong Jin An, Han Sun Kim, Byung-Sik Cho, Sunghyouk Park

**Affiliations:** 1 Natural Products Research Institute, College of Pharmacy, Seoul National University, Seoul, Republic of Korea; 2 Department of Biochemistry, College of Medicine, Inje University, Busan, Republic of Korea; 3 Department of Biochemistry, College of Medicine, Dongguk University, Gyeongju, Republic of Korea; 4 Department of Hematology, Catholic Hematology Hospital, Seoul St. Mary’s Hospital, College of Medicine, The Catholic University of Korea, Seoul, Republic of Korea

**Keywords:** 7 + 3 induction therapy, acute myeloid leukemia, F2R, predictive biomarkers, refractory response, TMEM150C

## Abstract

**Introduction:**

Acute myeloid leukemia (AML) is a heterogeneous hematologic malignancy with variable responses to standard 7 + 3 induction therapy. Although transcriptional profiling can capture biological phenotypes beyond genetic mutations, regimen-specific predictive biomarkers for 7 + 3 therapy have not been established due to methodological limitations in existing studies.

**Methods:**

To identify transcriptomic signatures for 7 + 3 response, we analyzed two ethnically diverse AML cohorts: OHSU BeatAML2.0 (n = 264) and SIH/JIH/ZIH AML (n = 582). Machine learning algorithms, including least absolute shrinkage and selection operator (LASSO), and cross-cohort intersection analysis were employed to identify candidate genes associated with 7 + 3 therapy outcomes. The identified candidates were further evaluated using qRT-PCR in an independent AML patient cohort (n = 58) and cytarabine sensitivity assays in AML cell lines.

**Results:**

Intersection analysis identified seven candidate genes showing concordant associations with refractory response in OHSU and poor overall survival in SIH/JIH/ZIH. In the independent clinical cohort, only 2 of the 7 candidates, *TMEM150C* and *F2R*, were reliably detectable, with significantly elevated expression in refractory patients (4.2-fold and 2.9-fold, respectively). AML cell lines with higher TMEM150C and F2R expression also exhibited reduced cytarabine sensitivity.

**Discussion:**

These findings from ethnically diverse cohorts, independent patient validation, and functional studies suggest that TMEM150C and F2R may serve as potential predictive biomarkers for 7 + 3 therapy response in AML, enabling personalized treatment selection.

## Introduction

Acute myeloid leukemia (AML) is a malignant neoplasm characterized by disrupted myeloid differentiation and accumulation of abnormal bone marrow blasts (1–3), with a five-year survival rate of only 27% for patients over 20 (4, 5). Furthermore, the heterogeneous molecular characteristics of AML, including diverse genetic alterations and variable gene expression patterns, represent a significant challenge affecting treatment outcome (6, 7). Therefore, understanding how these variations affect therapeutic response is essential for improving treatment strategies.

AML treatment consists of two main phases: induction therapy to achieve complete remission, followed by consolidation therapy to eliminate minimal residual AML cells and prevent relapse (8, 9). The 7 + 3 regimen has been the most common induction therapy, combining continuous cytarabine for seven days with anthracycline (daunorubicin or idarubicin) for three days (10, 11). However, 30-40% of patients show resistance to this regimen, with response variability driven by genetic, clinical, and treatment factors (12). This underscores the importance of developing predictive biomarkers to identify resistant patients early and to guide personalized therapeutic selection.

Various biomarker approaches have been explored to predict treatment response in AML. Genetic mutations such as *FLT3* internal tandem duplication (*FLT3*-ITD) and *NPM1* mutations are well-established molecular markers that influence risk stratification and treatment strategies in AML (13, 14). However, gene mutations alone have limited predictive power, as they fail to capture dynamic biological phenotypes reflected in transcriptional profiles (15, 16). Nevertheless, current gene expression studies are constrained by limited sample sizes, lack of external validation, and predominant focus on overall survival rather than regimen-specific efficacy. Therefore, validated biomarkers specifically for predicting the 7 + 3 response remain an unmet clinical need. Here, we sought to identify such biomarkers using large, ethnically diverse AML cohorts with independent clinical and cell-line validation.

## Materials and methods

### Acquisition of clinical data

Clinical data were obtained from two datasets: the OHSU BeatAML2.0 cohort (17) and the SIH/JIH/ZIH AML dataset published previously (18). The OHSU BeatAML2.0 cohort, comprising 942 patients, provided gene expression, survival, and induction therapy response data (http://www.vizome.org/aml). Gene expression data were presented in reads per kilobase per million mapped reads (RPKM) and were log2-transformed prior to analysis. In addition, we utilized an AML dataset from three centers: the Shanghai Institute of Hematology (SIH, n = 442), Jiangsu Institute of Hematology (JIH, n = 110), and Zhejiang Institute of Hematology (ZIH, n = 103), providing survival, induction therapy, and gene expression data in transcripts per million (TPM). Overall survival data were provided by the authors of the original study upon request. The original study reported a median follow-up of 20.2 months. The RNA-seq data are deposited in GSA-Human under accession HRA002693 (controlled access).

### Identification of differentially expressed genes based on induction therapy response in OHSU BeatAML2.0 dataset

Patients with documented 7 + 3 treatment consisting of cytarabine and idarubicin and available induction-response information were included in the analysis. DEG between complete remission (CR) and refractory (R) groups was analyzed by calculating fold change (FC; calculated by subtracting the median log2-transformed expression of CR from that of R patients) and false discovery rate (FDR; Benjamini–Hochberg method) for each gene. A total of 786 upregulated DEGs (log_2_FC > 0.6, FDR < 0.25) and 805 downregulated DEGs (log_2_FC < -0.6, FDR < 0.25) were identified. This relatively broad FDR threshold was used for initial feature screening to retain potentially informative candidates. Expression levels of these genes from 264 patients were used as input features for Least Absolute Shrinkage and Selection Operator (LASSO) regression performed in R (glmnet and caret packages). R and CR patients were coded as 1 and 0, respectively, for LASSO regression. 15 upregulated genes (coefficients > 0.01) and 11 downregulated genes (coefficients < −0.1) were selected. The dataset was divided into training and testing sets with a ratio of 70%/30%.

### The construction of clinical prediction models

The 26 selected genes were used as features in prediction models. Prediction models were built using machine learning algorithms including random forest, gradient boosting, and logistic regression with the scikit, pandas, and numpy packages in Python 3.9.13. Model performance and their predicted probabilities were assessed using Receiver Operating Characteristic (ROC) curves and Area Under the Curve (AUC) values.

### Survival analysis in SIH/JIH/ZIH AML dataset

Since the SIH/JIH/ZIH AML dataset did not provide induction therapy response data, overall survival was analyzed as a surrogate outcome. For survival analysis, status = 1 was treated as a death event and status = 0 as censored. After excluding 56 acute promyelocytic leukemia patients, 376 patients who received 7 + 3 therapy (IA induction: idarubicin plus cytarabine) were included in the survival analysis. Patients were stratified into high- and low-expression groups using an optimal cutoff determined with the surv_cutpoint function of the R package survminer, which determines the optimal cutoff based on maximally selected rank statistics. A minimum proportion of 0.20 was required in each group, and the resulting cutoff was applied using surv_categorize. Kaplan–Meier survival curves were compared using the log-rank test. The same survival analysis was performed on 206 patients who did not receive 7 + 3 combinational therapy but other forms of induction therapy. For genome-wide survival screening, Cox proportional hazards regression was performed for each gene, and the P values obtained across all tested genes were adjusted for multiple testing using the Benjamini–Hochberg method.

### Gene set enrichment analysis

GSEA was performed using Broad Institute software with log2-transformed fold changes and hazard ratios as input for OHSU and SIH/JIH/ZIH datasets, respectively, using the Reactome v2023 gene set. To assess the specificity of pathways associated with 7 + 3 therapy response, as opposed to those broadly associated with AML, GSEA was additionally performed using survival data from patients who received other induction regimens.

### Intersection of significant genes in both datasets

For the intersection analysis, a more stringent FDR threshold of < 0.05 was applied to both the OHSU BeatAML2.0 and SIH/JIH/ZIH AML. Upregulated candidates were defined as genes with log_2_FC > 0.6 (OHSU BeatAML2.0) and HR > 1.5 (SIH/JIH/ZIH AML); downregulated candidates as log_2_FC < -0.6 and HR < 0.667, respectively. Common genes were further cross-referenced with the 26 LASSO-selected genes.

### Internal cohort validation with qRT-PCR

Bone marrow samples were collected from AML patients before 7 + 3 induction therapy, with approval from the Institutional Review Board of The Catholic University of Korea (no. KC22TISI0110), in accordance with the Declaration of Helsinki, and written informed consent from all participants. The cohort comprised 58 samples (R, n = 28; CR, n = 30; **Supplementary Table S1**). Bone marrow mononuclear cells were isolated using Ficoll paque plus and cryopreserved in freezing buffer (Horse serum: DMSO = 9:1) at -80 °C. Total RNA was extracted using the Total RNA Extraction Kit (iNtRON Biotechnology, Seongnam, Korea), and cDNA was synthesized using the High-Capacity cDNA Reverse Transcription Kit (Applied Biosystems, CA, USA) per the manufacturer’s instructions. qPCR was performed using iTaq Universal SYBR Green Supermix (Bio-Rad, CA, USA) on a QuantStudio 5 Real-Time PCR instrument (Thermo Fisher Scientific, MA, USA). The primers used are listed in **Supplementary Table S2**. Data were normalized using *GAPDH*, and Relative Quantification (RQ) values were calculated using the formula 2^-ΔΔCt.

### Cell culture and cell viability

Kasumi-1 cells were authenticated by short tandem repeat (STR) profiling, confirming 100% match to Kasumi-1 (CRL-2724). U937 (CRL-1593.2) cells were purchased from ATCC (ATCC, VA, USA). All cell lines were confirmed to be mycoplasma-free using the mycoplasma detection kit (Biomax) prior to experiments. Cells were cultured in RPMI 1640 media supplemented with fetal bovine serum (FBS; 20% for Kasumi-1, 10% for U937), 100 U/mL penicillin, and 100 μg/mL streptomycin, at 37 °C in a 5% CO_2_ incubator. Cell viability was measured using CCK-8 assay after cytarabine treatment for 72 h.

### Statistical analysis

All cell line experiments were performed independently at least three times. Data are presented as mean ± standard error of the mean (SEM). Statistical analyses included log-rank test, Mann-Whitney U test, Welch’s t-test, and Student’s *t*-test using GraphPad Prism 9.1.1 (GraphPad Software, CA, USA) or R (version 4.2.3), with the optimal cut-off value for Kaplan-Meier analysis determined by the surv_cutpoint method. A *p*-value < 0.05 was considered statistically significant.

## Results

### Overview of the analytical approach for identifying expression signatures for 7 + 3 therapy response

To identify transcriptomic candidates associated with 7 + 3 therapy outcomes in AML, we employed a stepwise analytical approach using two ethnically distinct large-scale datasets (OHSU BeatAML2.0 and SIH/JIH/ZIH AML), as illustrated in **Figure 1**. The workflow consisted of initial feature screening and LASSO-based selection in OHSU, followed by more stringent cross-cohort intersection analysis to prioritize candidate genes. This approach integrated direct treatment response data from OHSU with complementary survival outcome information from the SIH/JIH/ZIH AML cohort to identify candidates showing consistent associations across diverse populations. Identified candidates were further validated in an independent clinical cohort and AML cell lines.

**Figure 1:**
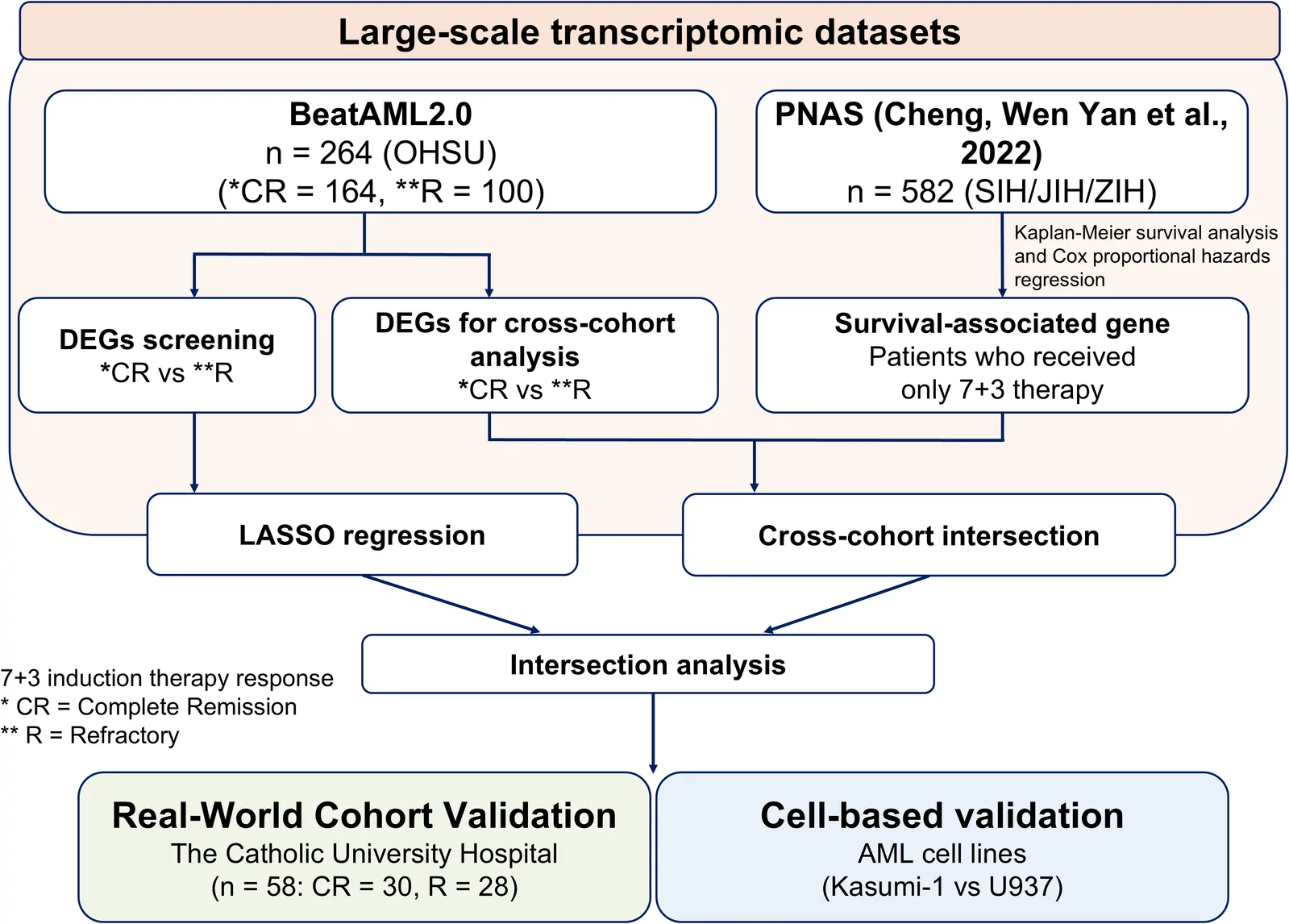
The workflow of this study. The OHSU BeatAML2.0 database categorizes 7 + 3 therapy response as CR and R. Among differentially expressed genes (DEGs) between the two groups (log_2_FC > 0.6 or < -0.6 and FDR < 0.25), the key genes were selected using the LASSO machine learning model. In SIH/JIH/ZIH AML database, survival-related genes of patients treated with only 7 + 3 therapy were analyzed (HR > 1.5 or < 0.667 and FDR < 0.05). By intersection analysis (log_2_FC > 0.6 or < -0.6 and FDR < 0.05 for OHSU BeatAML2.0 database & HR > 1.5 or < 0.667 and FDR < 0.05 for SIH/JIH/ZIH AML database), gene signature reflecting both response and survival outcome was identified and then validated in our internal AML cohort. Further validation was performed in AML cell lines with differential cytarabine sensitivity. CR, Complete Remission group; R, Refractory group.

### Identification of genes associated with response to 7 + 3 induction therapy in OHSU BeatAML2.0 dataset

We first analyzed 264 patients from the OHSU BeatAML2.0 database treated with 7 + 3 induction therapy, with responses directly classified as complete remission (CR) or refractory (R), and performed DEG analysis to identify transcriptomic differences between groups. The top 20 up- and downregulated genes are presented in **Supplementary Tables S3**, **S4**, respectively. Among these, *DSG2* (Desmoglein 2) was the most upregulated gene (**Figure 2A**). It is a cell adhesion protein associated with tumorigenesis and metastasis in breast, lung, and colon cancers (19, 20), and known to maintain the undifferentiated state of hematopoietic cells (21). *NKX2-3* (NK2 Homeobox 3), expressed in hematopoietic stem cells, showed the most significant downregulation in treatment-resistant patients (**Figure 2B**). Its aberrant activation has been shown to inhibit megakaryocytic-erythroid differentiation in AML (22), although its broader role in hematological malignancies remains context-dependent (23, 24). These findings suggest that our DEG analysis identified genes with established relevance to hematopoietic differentiation and cancer-related processes, supporting the validity of our approach.

**Figure 2:**
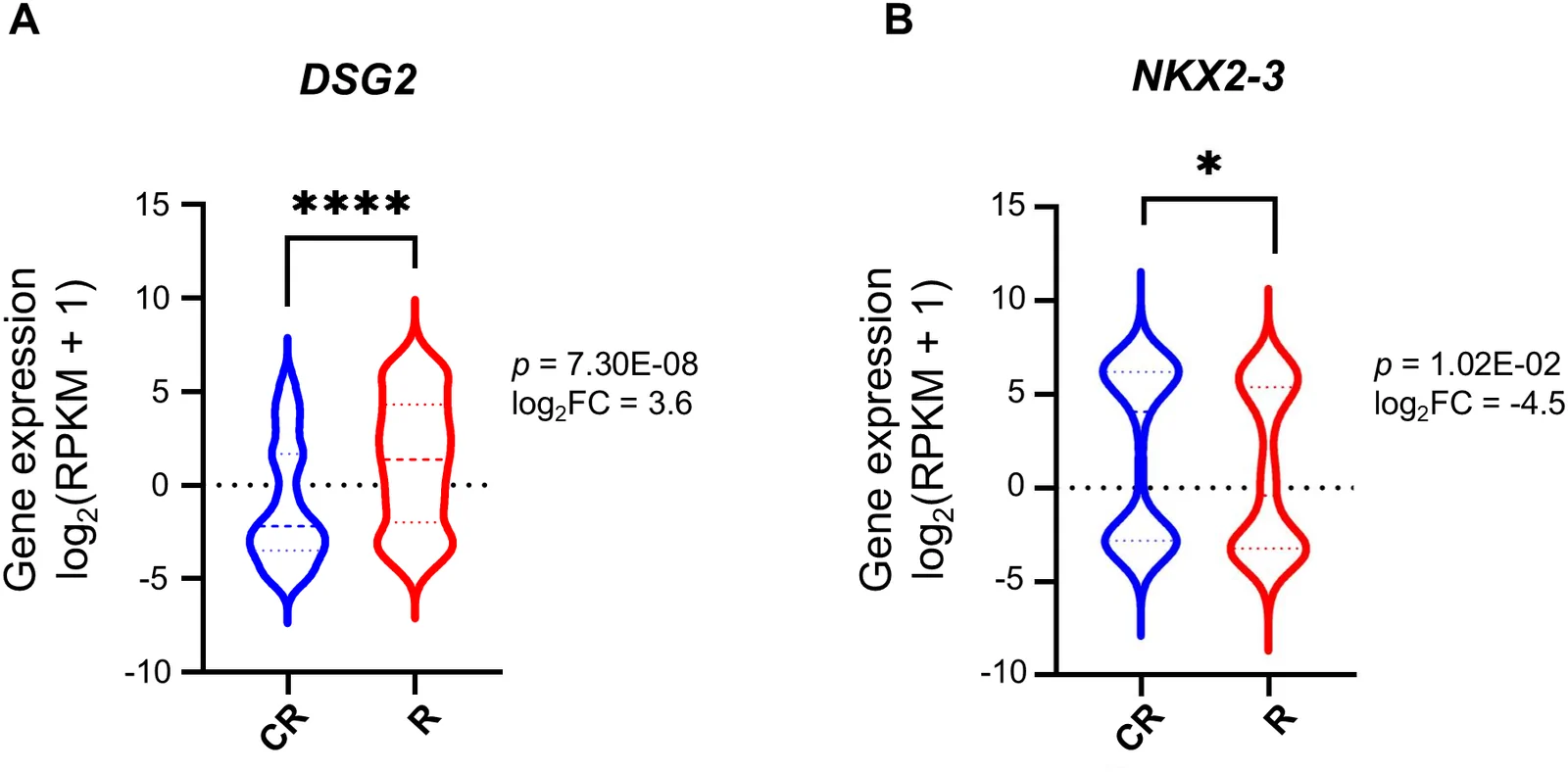
Representative genes are significantly associated with poor response to induction therapy. The difference in log_2_FC of gene expression was compared between R and CR groups. **(A)** *DSG2*, the most upregulated gene in refractory patients. **(B)** *NKX2-3*, the most downregulated gene excluding pseudogenes in refractory patients. Log_2_ fold change (log_2_FC) was calculated as median (R) - median (CR) from log_2_-transformed expression values. Statistical significance was determined using Mann-Whitney U test. *P < 0.05, ****P < 0.0001 vs. CR.

### Prediction of the therapy outcome with machine learning

We next applied LASSO regression to prioritize informative features from the broadly screened response-associated DEGs. After classifying the DEGs as upregulated (log_2_FC > 0.6) or downregulated (log_2_FC < -0.6), we performed the LASSO regression analysis (**Supplementary Figure S1**). From the 786 upregulated features in refractory patients, we identified 15 genes with coefficients > 0.01 (**Supplementary Figures S1A, B**, **Supplementary Table S5**), including *HMGA2* (High Mobility Group AT-Hook 2), known to contribute to therapy resistance (25). Similarly, 11 downregulated genes with coefficients < -0.1 were identified (**Supplementary Figures S1C, D**, **Supplementary Table S6**), including *TFR2* (Transferrin Receptor 2), whose high expression is associated with better prognosis in AML (26). To further evaluate the predictive information of these 26 gene signatures, we evaluated their predictive capacity using multiple machine learning algorithms, including random forest, gradient boosting, and logistic regression. Random forest and gradient boosting consistently identified the genes selected by LASSO (**Figures 3A, C**) and achieved high predictive performance, with AUC values of 0.92 and 0.85, respectively (**Figures 3B, D**). Logistic regression also demonstrated strong predictive ability, with an AUC of 0.87 (**Figure 3E**). These results support the predictive capacity of the LASSO-selected gene set across different modeling approaches.

**Figure 3:**
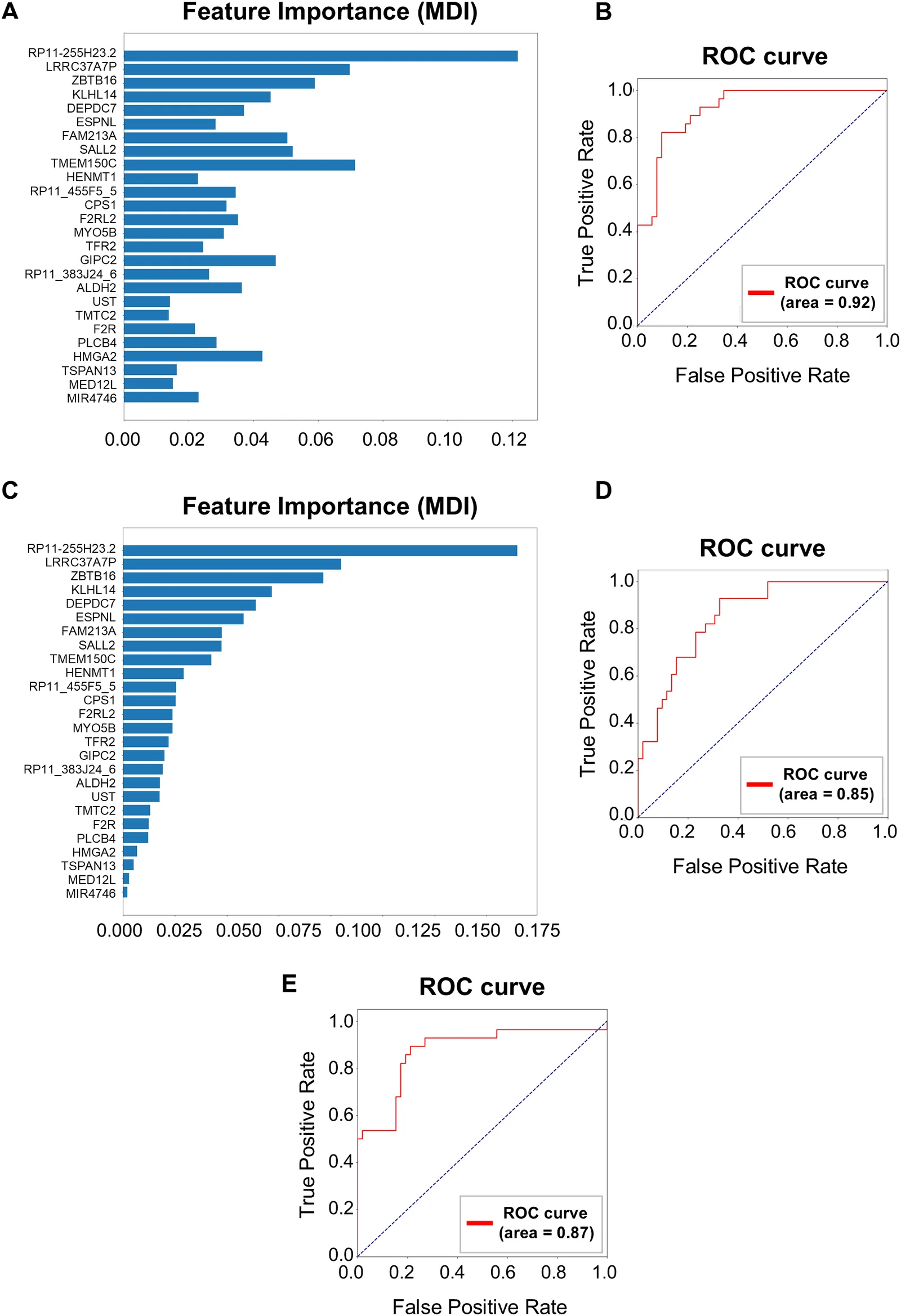
Robust predictive performance from a variety of machine learning algorithms. **(A)** Feature importance plots of random forest method for 26 genes determined by LASSO. **(B)** ROC curve of the random forest. AUC = 0.92. **(C)** Feature importance plots of gradient boosting for 26 genes determined by LASSO. **(D)** ROC curve of the gradient boosting. AUC = 0.85. **(E)** ROC curve of the logistic regression. AUC = 0.87. MDI, mean decrease in impurity; ROC, receiver operating characteristic.

### Identification of genes associated with poor overall survival in SIH/JIH/ZIH AML dataset

We next analyzed a transcriptomic dataset from three Chinese centers to validate our findings in an ethnically distinct population. Since direct therapy response data were unavailable, overall survival was used as a surrogate outcome. Using Kaplan-Meier survival analysis and Cox proportional hazards regression, we identified genes associated with poor overall survival in patients who received the 7 + 3 regimen. The top 20 genes with highest hazard ratio (HR) are listed in **Supplementary Table S7**. In parallel, the same analysis was performed on patients receiving alternative therapies (**Supplementary Table S8**). Genes identified in both analyses were excluded to focus on those specific to 7 + 3 therapy. **Supplementary Table S9** lists the top 20 genes with the highest HR values associated with poor survival specifically in the 7 + 3 treatment group. The top-ranked genes, *PVR* and *ARHGEF16*, showed the highest hazard ratios (HR = 4.36 and 4.29, respectively) (**Supplementary Figure S2**). *PVR* is an immune checkpoint signaling molecule (27, 28), and *ARHGEF16* is a regulator of cell migration (29), both of which have established associations with AML prognosis and related phenotypes. These findings suggest that our approach should be relevant for the identification of the predictive indicators for AML treatment.

### Biological coherence across datasets supporting integrated gene identification for 7 + 3 therapy response

To determine whether the two datasets showed consistent biological patterns associated with 7 + 3 therapy outcomes, we performed GSEA analysis. It revealed that both sets of data share common pathways associated with refractory response. In particular, we observed a common increase in pathways related to blood coagulation and cellular adhesion (**Supplementary Tables S10**, **11**). In addition, both datasets showed a predominant downregulation of immune function-related pathways (**Supplementary Tables S12** and **13**). These findings validate the biological coherence between cohorts, supporting integrated biomarker identification and providing mechanistic insights.

### Identification of 7 common genes associated with refractory response

To identify robust gene candidates associated with poor response, we conducted an intersection analysis across the two ethnically distinct datasets. We first compiled lists of significantly upregulated genes in OHSU BeatAML2.0 (log_2_FC > 0.6, FDR < 0.05) and genes whose high expression was associated with poor survival in the SIH/JIH/ZIH AML dataset (HR > 1.5, FDR < 0.05). Similarly, we identified downregulated genes in OHSU BeatAML2.0 and genes whose low expression was associated with poor survival in the SIH/JIH/ZIH AML dataset (log_2_FC < -0.6, HR < 0.667, and FDR < 0.05). This analysis resulted in 105 common upregulated genes and 15 common downregulated genes. These were further intersected with the 26 LASSO-selected genes from OHSU BeatAML2.0. As a result, we identified 7 candidate genes showing concordant associations with refractory response in OHSU BeatAML2.0 and poor survival in the SIH/JIH/ZIH cohort (**Supplementary Figure S3**). Among these, 5 genes (*TMEM150C*, *F2R*, *PLCB4*, *CPS1*, and *GIPC2*) exhibited increased expression levels in refractory patients and were associated with poorer survival (**Figures 4A-E**, **Supplementary Figures S4A-E**). On the other hand, low expression of *ZBTB16* and *ESPNL* was associated with diminished response to 7 + 3 therapy (**Figures 4F, G**; **Supplementary Figures S4F, G**). Collectively, the identification of these 7 genes represents an important finding for 7 + 3 therapy response prediction.

**Figure 4:**
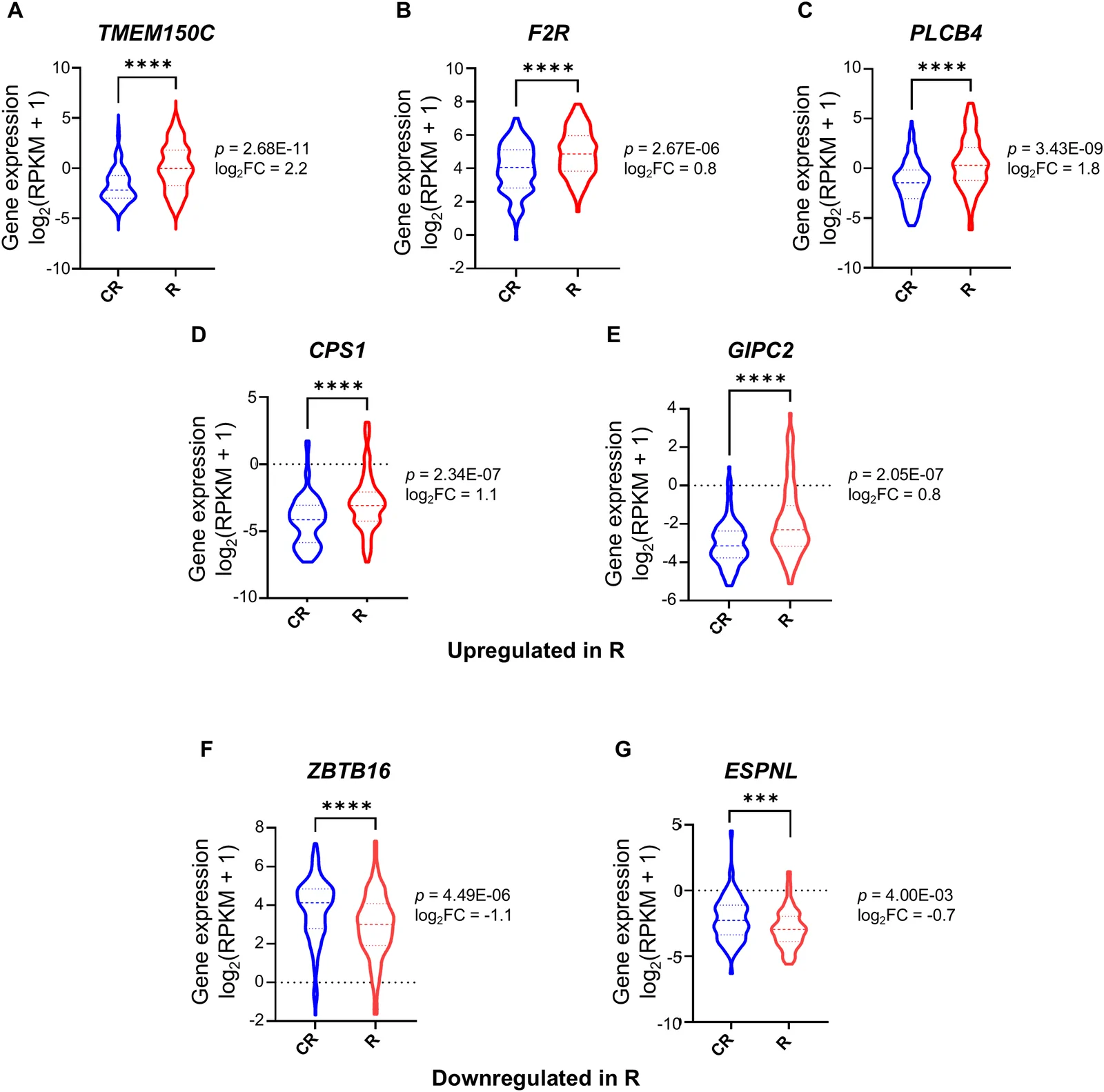
Gene expression of 7 gene signatures between two therapy response groups in OHSU BeatAML2.0. **(A-G)** Log_2_FC was calculated as the difference between the median log_2_-transformed expression levels of the R and CR groups. Genes in **(A–E)** were upregulated in R patients, whereas those in **(F, G)** were downregulated in R patients. Statistical significance was determined using Mann-Whitney U test. ***P < 0.001, ****P < 0.0001 vs. CR.

### Independent validation of 7 + 3 therapy response genes in real-world AML patients

To evaluate the clinical relevance of the 7 genes, expression patterns were examined in our independent AML cohort (R, n = 28; CR, n = 30) using qRT-PCR. Out of the 7 genes, 5 genes (*CPS1*, *GIPC2*, *PLCB4*, *ESPNL*, *ZBTB16*) demonstrated Ct values higher than 30, indicating very low basal expression levels that preclude reliable quantification of differential expression between the CR and R groups. Therefore, we focused on *F2R* and *TMEM150C* genes, which showed measurable expression levels. Our validation revealed a notable increase in the expression levels of *TMEM150C* (4.2-fold) and *F2R* (2.9-fold) in refractory AML patients compared to the CR group (**Figures 5A, B**). These results are consistent with our bioinformatic findings, confirming *F2R* and *TMEM150C* as measurable predictive markers in clinical samples. To further explore the biological context of these two candidates, we examined their correlations with GSEA core genes from the fibrin clotting and elastic fiber formation pathways enriched in both the OHSU BeatAML2.0 and SIH/JIH/ZIH AML cohorts. Multiple core genes showed significant, directionally concordant correlations with *F2R* or *TMEM150C* in both cohorts (**Supplementary Figures S5A, B**). *TMEM150C* also showed positive correlations with *F2R* and several fibrin clotting core genes (**Supplementary Figure S5B**). These findings support associations of the two clinically detectable candidates with coagulation- and ECM-related transcriptional programs identified across the two discovery cohorts.

**Figure 5:**
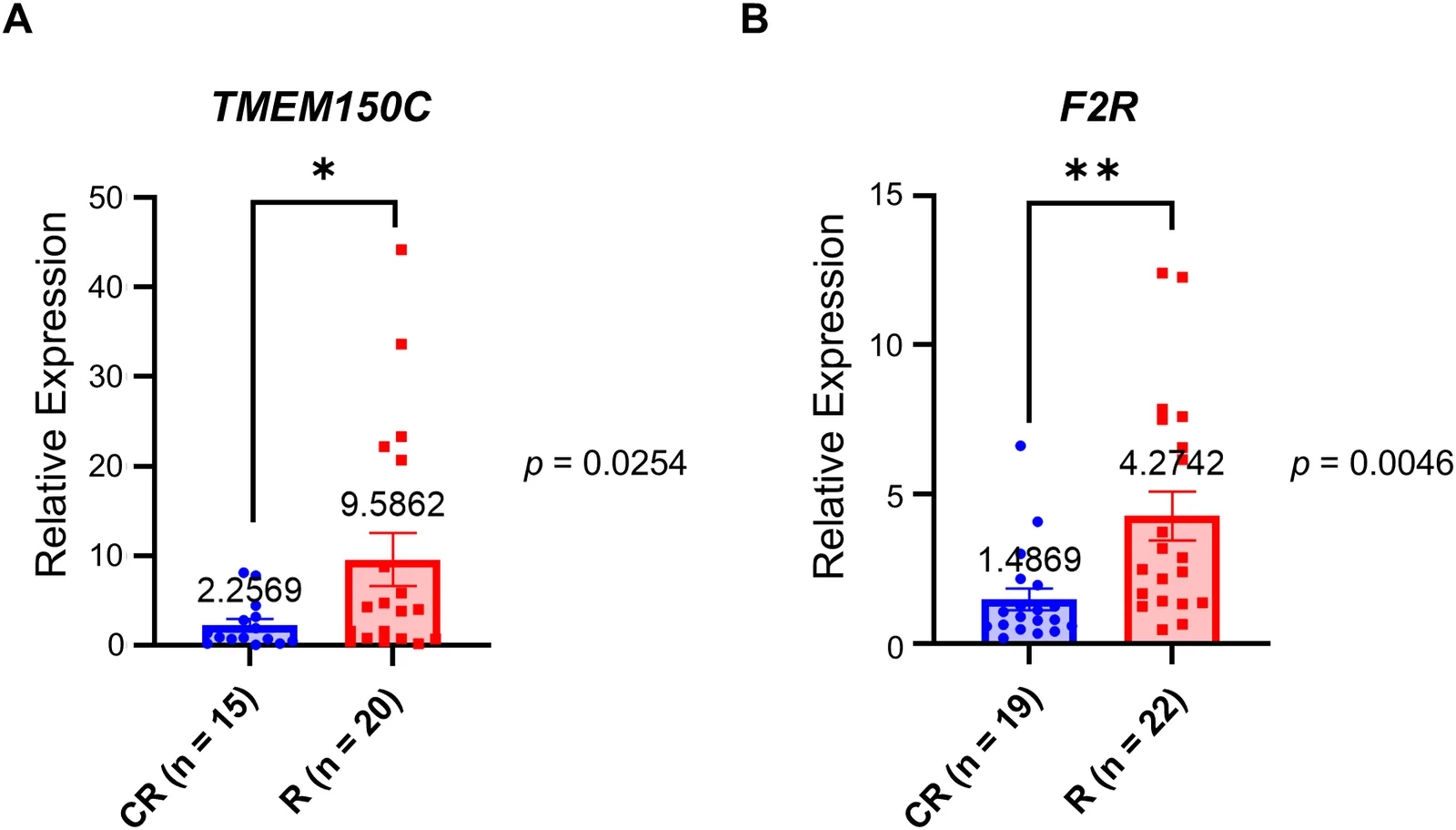
*TMEM150C* and *F2R* genes as expression markers of refractory patients in independent AML cohort. mRNA expression levels of *TMEM150C* **(A)** and *F2R* **(B)** in the CR and R groups measured by qRT-PCR. Data were normalized to *GAPDH* and represent mean ± SEM relative to the CR group. Statistical significance was determined using Welch’s t-test. *P < 0.05, **P < 0.01 vs. CR.

### Experimental validation of *TMEM150C* and *F2R* correlation with reduced cytarabine sensitivity in AML cells

Finally, we investigated whether *TMEM150C* and *F2R* expression is associated with cytarabine resistance, the primary component of 7 + 3 chemotherapy. U937 and Kasumi-1 cells were selected based on their well-characterized differences in cytarabine sensitivity, with U937 being sensitive and Kasumi-1 insensitive (30, 31). Consistent with these findings, our results also demonstrated reduced drug responsiveness in Kasumi-1 cells compared to U937 cells, as evidenced by a 2-fold higher half-maximal inhibitory concentration (IC_50_) value (**Figure 6A**). The relative expression of both genes was significantly higher in Kasumi-1 cells than in U937 cells (**Figure 6B**). These results demonstrated that higher expression of both genes is associated with decreased cytarabine sensitivity, further supporting *TMEM150C* and *F2R* as potential predictive biomarkers for 7 + 3 therapy response.

**Figure 6:**
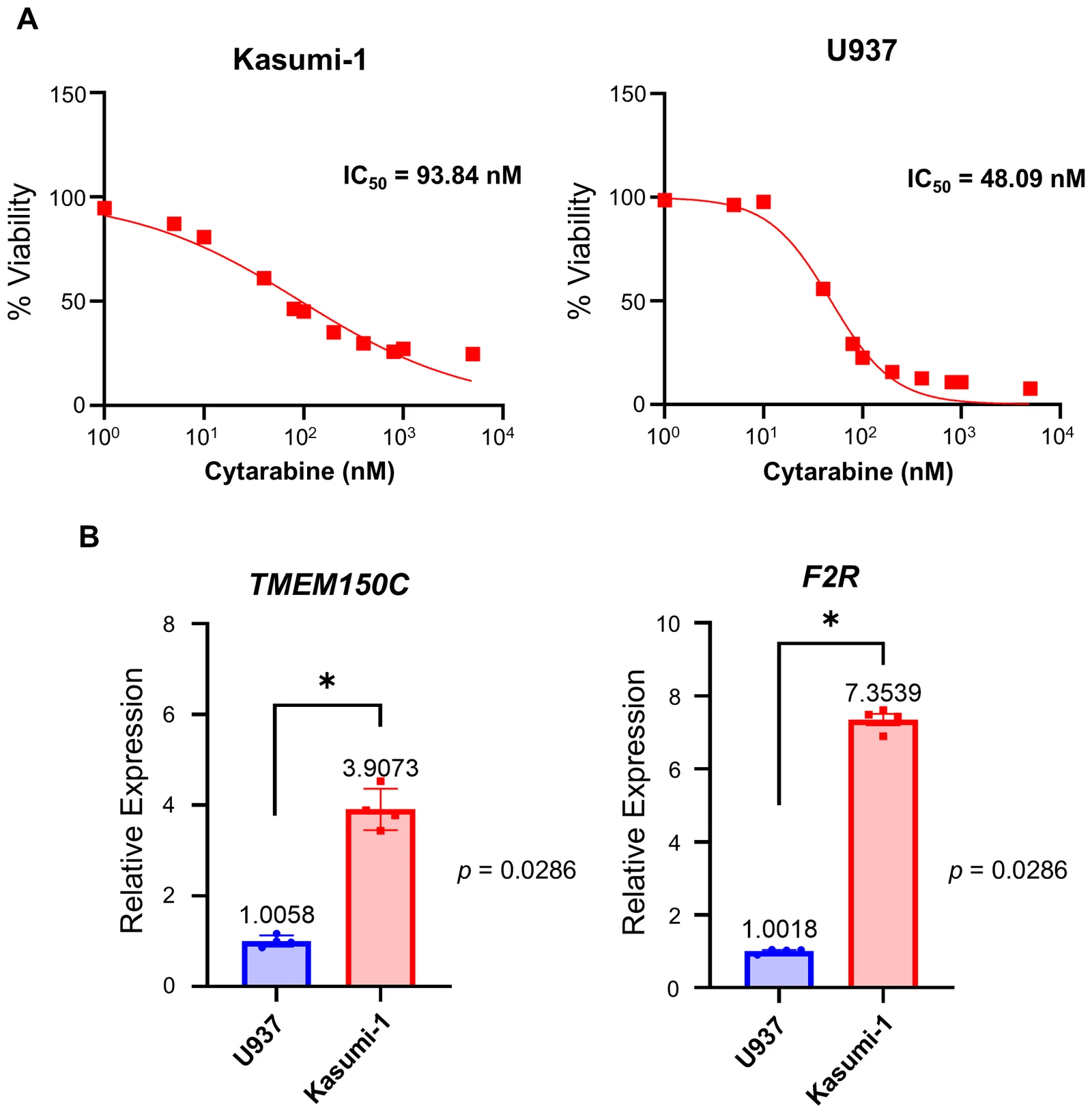
High expression of *TMEM150C* and *F2R* genes is associated with decreased sensitivity to cytarabine. **(A)** The IC_50_ values for cytarabine in Kasumi-1 and U937 cells. **(B)** The expression levels of *TMEM150C* and *F2R* were evaluated using qRT-PCR and normalized to *GAPDH*. Data represent mean ± SEM relative to U937 cells from at least three independent experiments. Statistical significance was determined using Mann–Whitney U test. ***P < 0.001 vs. U937 cells.

## Discussion

AML is a highly heterogeneous disease with diverse genetic and molecular subtypes that significantly affect treatment response. Despite the widespread use of the 7 + 3 regimen, robust predictive biomarkers for this regimen remain limited. Therefore, this study aimed to identify expression signatures associated with 7 + 3 therapy resistance and poor prognosis in AML, leading to the identification of *TMEM150C* and *F2R* as novel biomarkers through analysis of ethnically diverse cohorts and experimental validation.

Previous studies have focused on identifying molecular markers to predict chemotherapy response. Gene expression-based studies demonstrated that MYC, mitochondrial OXPHOS, mTOR, and stemness-related expression signatures are key determinants of 7 + 3 therapy resistance (32), and others have used refractory patient signatures as prognostic classifiers to predict survival outcome (16, 17, 33, 34). Additionally, mutation-based studies have utilized gene variant patterns to predict drug sensitivity (35). While our study shares these fundamental goals, our analytical approach extends previous studies in several important ways. First, we utilized large cohorts comprising 846 patients (total) across two transcriptomic datasets, providing enhanced statistical power; the identification of known resistance-related genes such as *HMGA2* further supports the reliability of our approach (36). Second, we integrated both treatment response and long-term survival data, ensuring that identified markers reflect not only initial therapy failure but also sustained poor prognosis. Third, most importantly, we conducted comprehensive experimental validation in independent clinical samples and cell lines, which is rarely performed in computational studies. This validation revealed that only 2 of 7 candidate genes (*TMEM150C* and *F2R*) demonstrated reliable detectability, highlighting the critical importance of experimental confirmation in real clinical samples. These findings further suggest that clinical detectability should be considered early in candidate screening in future biomarker studies to improve their potential for clinical use. Finally, our multi-ethnic validation strengthens the generalizability of identified biomarkers.

The seven candidate genes have been associated with diverse biological pathways, including epigenetic regulation, signal transduction, metabolic reprogramming, and tumor microenvironment interactions in AML and other cancers (**Supplementary Table S14**) (37–40), providing insights into potential resistance mechanisms. *CPS1* and *PLCB4*, both upregulated in refractory patients, are associated with cytarabine resistance via pyrimidine biosynthesis upregulation (41) and multidrug-resistant leukemia cells (42), respectively. Among these, *F2R* and *TMEM150C* warrant particular attention as experimentally validated markers. The previously reported functions of *F2R* provide biological support for its association with poor treatment response. *F2R*, also known as PAR-1, is a thrombin receptor involved in platelet activation and coagulation. In our study, *F2R* showed concordant correlations with core genes of the fibrin clotting pathway, which was among the top upregulated GSEA pathways in both the OHSU BeatAML2.0 and SIH/JIH/ZIH AML cohorts, consistent with its established coagulation-related function. F2R has also been implicated in tumor progression in gastric adenocarcinoma, where its knockdown reduced cancer-cell proliferation, migration, and invasion. Its correlations with elastic fiber/ECM core genes in both AML cohorts further suggest a broader ECM-related transcriptional association. In AML, *F2R*/PAR-1 has been reported to promote leukemic stem cell growth under low-cell-density conditions, potentially contributing to disease persistence and treatment resistance (43–45). In contrast, *TMEM150C* remains functionally uncharacterized in AML. In our analysis, *TMEM150C* showed concordant correlations with elastic fiber core genes and positive correlations with *F2R* across both cohorts. The overlapping correlation patterns of *TMEM150C* and *F2R* suggest that the two candidates may be linked to a partially shared ECM/coagulation-associated transcriptional program. These findings provide a possible biological context for *TMEM150C* and support it as a candidate for further mechanistic investigation.

While this study presents a novel approach to therapeutic predictive biomarker discovery, we acknowledge several limitations. First, because induction therapy response information was unavailable in the Chinese AML dataset, overall survival was used as a surrogate marker, which may introduce some inaccuracies compared to direct response assessment. Second, although *TMEM150C* and *F2R* expression was associated with cytarabine sensitivity in AML cell lines, the comparison between Kasumi-1 and U937 cells does not establish a causal relationship, as these cell lines differ in multiple genetic and molecular characteristics that may independently influence drug sensitivity. Further functional studies using genetic perturbation of *TMEM150C* and *F2R* are therefore required to determine their functional contribution to cytarabine resistance and potential roles as therapeutic targets. In conclusion, this study identifies *TMEM150C* and *F2R* as potential biomarkers associated with 7 + 3 therapy response through integrated transcriptomic analysis and experimental validation, providing a basis for further investigation of their potential utility in personalized AML treatment.

## Data Availability

Data from the independent 58-patient cohort are available from the corresponding author upon reasonable request.
