# Supplementary material for: TMEM150C and F2R as transcriptomic signatures for refractoriness to 7 + 3 induction therapy in acute myeloid leukemia
Source: Front Oncol. 2026 Oct 23;16:1926332. doi: 10.3389/fonc.2026.1926332 (PMC13739514; doi:10.3389/fonc.2026.1926332)
Supplement: Supplementary file 1 [file DataSheet1.docx]

**Supplementary figures and Tables**

**Supplementary Table 1. Patient characteristics**

| **Variable** | | **Total (N = 58)** | **CR (N = 30)** | **R (N = 28)** |
| --- | --- | --- | --- | --- |
| Sex, n (%) | Women | 26 (45%) | 17 (57%) | 9 (32%) |
|  | Men | 32 (55%) | 13 (43%) | 19 (68%) |
| Age | Median (range) | 49 (17-70) | 44 (19-68) | 53.5 (17-70) |
| Type, n (%) | de novo | 53 (91%) | 30 (100%) | 23 (82%) |
|  | Myelodysplasia-related | 4 (7%) |  | 4 (14%) |
|  | Therapy-related | 1 (2%) |  | 1 (4%) |
| ELN risk, n (%) | Favorable | 23 (40%) | 21 (70%) | 2 (7%) |
|  | Intermediate | 13 (22%) | 5 (17%) | 8 (29%) |
|  | Adverse | 22 (38%) | 4 (13%) | 18 (64%) |
| FLT3-ITD, n (%) | Positive | 12 (21%) | 2 (7%) | 10 (36%) |
|  | Negative | 46 (79%) | 28 (93%) | 18 (64%) |
| NPM1 mutation, n (%) | Positive | 9 (16%) | 6 (20%) | 3 (11%) |
|  | Negative | 49 (84%) | 24 (80%) | 25 (89%) |
| WBC (/μL) | Median [IQR] | 11.3 [4.5-27.7] | 11.3 [6.4-22.3] | 11.5 [3.6-55.3] |
| Bone marrow blasts (%) | Median [IQR] | 75 [57.3-90] | 72.5 [62.3-88.5] | 78 [55-90] |
| Status, n (%) | Alive | 26 (45%) | 20 (67%) | 6 (21%) |
|  | Dead | 32 (55%) | 10 (33%) | 22 (79%) |

**Supplementary Table 2. Primer sequences**

| **Gene** | **Reverse (5' -> 3')** | **Forward (5' -> 3')** |
| --- | --- | --- |
| *TMEM150C* | AGGTCAAGGAAGTTCCGACG | GCCCTTGTGGTAGCTGTTCT |
| *F2R* | AGCTGATCTTAAAGGGGAGCA | CACCGGAGTGTTTGTAGTCAG |
| *PLCB4* | TTCATAACCATGCGAGTTCGG | GATGGTGTTATTGCAGCCACT |
| *CPS1* | TGCTTTAAGCCCACCTCATTG | GGATCAGGAGGTCTGTCCATT |
| *GIPC2* | GTCAAGTGCCACAGCAAATTC | ATGCCTTCTGAAACCAAAGCA |
| *ZBTB16* | TCATCTCCGTCTTCACCTCAG | TGCAGTGGACAGTTTGATGAC |
| *ESPNL* | CACCAAGAACTTGACGCAGT | CATGAGTCGCCACTGAGAGC |
| *GAPDH* | GAGTCAACGGATTTGGTCGT | TTGATTTTGGAGGGATCTCG |

**Supplementary Table 3. Top 20 genes with higher expression in refractory patients in OHSU BeatAML2.0 database**

| **Genes** | **log_2_FC** | ***P*** | **FDR** |
| --- | --- | --- | --- |
| *DSG2* | 3.58 | 1.19E-07 | 3.98E-05 |
| *HMGA2* | 3.54 | 1.30E-08 | 8.61E-06 |
| *BEND4* | 3.51 | 1.49E-05 | 9.97E-04 |
| *LHX6* | 3.19 | 7.74E-08 | 2.82E-05 |
| *CDC42BPA* | 3.12 | 3.60E-08 | 1.68E-05 |
| *DPPA4* | 3.02 | 3.25E-04 | 7.68E-03 |
| *GPR173* | 2.97 | 6.17E-05 | 2.60E-03 |
| *DNTT* | 2.96 | 1.35E-05 | 9.26E-04 |
| *AJ006998.2* | 2.92 | 9.88E-08 | 3.42E-05 |
| *MYCT1* | 2.92 | 1.18E-08 | 8.61E-06 |
| *EIF1AY* | 2.8 | 9.68E-03 | 0.068936 |
| *CHRDL1* | 2.79 | 8.25E-07 | 0.000139 |
| *TRO* | 2.73 | 8.69E-08 | 3.10E-05 |
| *PLSCR4* | 2.63 | 1.72E-06 | 2.35E-04 |
| *BAALC* | 2.62 | 2.27E-07 | 5.80E-05 |
| *TTTY14* | 2.6 | 2.40E-01 | 0.474763 |
| *IL12RB2* | 2.57 | 2.29E-07 | 5.80E-05 |
| *FAM213A* | 2.55 | 1.92E-08 | 1.05E-05 |
| *CD34* | 2.54 | 3.42E-07 | 7.58E-05 |
| *S100A16* | 2.54 | 3.11E-05 | 1.66E-03 |

FC, fold change; FDR, false discovery rate **Supplementary Table 4. Top 20 genes with lower expression in refractory patients in OHSU BeatAML2.0 database**

| **Genes** | **log_2_FC** | ***P*** | **FDR** |
| --- | --- | --- | --- |
| *NKX2-3* | -4.49 | 1.03E-02 | 7.19E-02 |
| *XXbacB444P24.8* | -3.63 | 5.12E-07 | 1.00E-04 |
| *RP11-129J12.2* | -3.62 | 2.17E-02 | 1.14E-01 |
| *IGKV2-23* | -3.11 | 8.32E-05 | 3.12E-03 |
| *UGGT2* | -3.08 | 6.37E-05 | 2.63E-03 |
| *LINC00900* | -2.77 | 9.22E-08 | 3.24E-05 |
| *IGKV1-22* | -2.69 | 3.19E-04 | 7.59E-03 |
| *HOXA11* | -2.69 | 3.60E-03 | 3.70E-02 |
| *RP11-388P9.2* | -2.54 | 5.66E-03 | 4.90E-02 |
| *COL19A1* | -2.53 | 3.94E-08 | 1.70E-05 |
| *AC091814.2* | -2.53 | 1.60E-01 | 3.78E-01 |
| *COL4A5* | -2.37 | 8.56E-02 | 2.62E-01 |
| *PRSS21* | -2.29 | 2.78E-02 | 1.33E-01 |
| *LTK* | -2.24 | 1.06E-08 | 8.61E-06 |
| *MAPK15* | -2.12 | 1.36E-08 | 8.61E-06 |
| *TCL1A* | -2.12 | 4.12E-06 | 4.20E-04 |
| *KIAA1456* | -2.08 | 1.59E-03 | 2.17E-02 |
| *CSMD1* | -2.07 | 1.31E-04 | 4.16E-03 |
| *GTSF1* | -2.05 | 3.76E-03 | 3.79E-02 |
| *MS4A1* | -2.04 | 9.21E-06 | 6.87E-04 |

FC, fold change; FDR, false discovery rate

**Supplementary Figure 1. LASSO analysis for genes with higher expression (A and B) and lower expression (C and D) in refractory patients. A and C** Misclassification error. **B and D** The lambda coefficient of L1 regularization.

**Supplementary Table 5. 15 highly expressed genes in refractory patients, as identified by LASSO analysis**

| **Genes** | **log_2_FC** | ***P*** | **FDR** |
| --- | --- | --- | --- |
| *HMGA2* | 3.54 | 1.30E-08 | 8.61E-06 |
| *FAM213A* | 2.55 | 1.92E-08 | 1.05E-05 |
| *TMEM150C* | 2.16 | 8.65E-11 | 6.26E-07 |
| *RP11-255H23.2* | 2 | 7.88E-10 | 1.50E-06 |
| *PLCB4* | 1.75 | 7.00E-09 | 7.27E-06 |
| *TSPAN13* | 1.66 | 1.93E-08 | 1.05E-05 |
| *SALL2* | 1.25 | 1.41E-09 | 2.15E-06 |
| *F2RL2* | 1.23 | 7.07E-06 | 5.83E-04 |
| *MED12L* | 1.23 | 4.89E-06 | 4.67E-04 |
| *ALDH2* | 1.19 | 6.62E-08 | 2.52E-05 |
| *CPS1* | 1.07 | 3.52E-07 | 7.73E-05 |
| *LRRC37A7P* | 0.98 | 1.68E-07 | 4.84E-05 |
| *GIPC2* | 0.84 | 3.12E-07 | 7.05E-05 |
| *F2R* | 0.82 | 3.51E-06 | 3.85E-04 |
| *HENMT1* | 0.61 | 1.09E-05 | 7.88E-04 |

FC, fold change; FDR, false discovery rate

**Supplementary Table 6. 11 lowly expressed genes in refractory patients, as identified by LASSO analysis**

| **Genes** | **log_2_FC** | ***P*** | **FDR** |
| --- | --- | --- | --- |
| *RP11-455F5.5* | -0.61 | 5.33E-06 | 4.87E-04 |
| *TFR2* | -0.62 | 2.85E-02 | 1.35E-01 |
| *TMTC2* | -0.63 | 1.85E-02 | 1.04E-01 |
| *ESPNL* | -0.68 | 4.59E-04 | 9.68E-03 |
| *RP11-383J24.6* | -0.76 | 8.69E-06 | 6.62E-04 |
| *UST* | -0.76 | 3.35E-04 | 7.87E-03 |
| *DEPDC7* | -0.81 | 2.41E-03 | 2.85E-02 |
| *MIR4746* | -0.86 | 9.56E-05 | 3.43E-03 |
| *MYO5B* | -0.96 | 4.62E-07 | 9.17E-05 |
| *ZBTB16* | -1.13 | 5.75E-06 | 5.15E-04 |
| *KLHL14* | -1.5 | 4.40E-07 | 8.90E-05 |

FC, fold change; FDR, false discovery rate

**Supplementary Table 7. Top 20 genes in order of Hazard Ratio for patients who received 7+3 therapy in SIH/JIH/ZIH AML dataset**

| **Genes** | **HR** | ***P*** | **FDR** |
| --- | --- | --- | --- |
| *SYTL4* | 4.44 | 2.23E-13 | 3.82E-09 |
| *PVR* | 4.43 | 1.44E-06 | 2.59E-04 |
| *ARHGEF16* | 4.29 | 1.87E-05 | 1.27E-03 |
| *CLU* | 4.24 | 2.61E-09 | 5.58E-06 |
| *CEACAM19* | 4.08 | 1.05E-06 | 2.21E-04 |
| *PHGDH* | 3.98 | 1.28E-05 | 1.02E-03 |
| *CPNE8* | 3.95 | 2.44E-08 | 2.19E-05 |
| *EPS8L1* | 3.90 | 5.24E-04 | 9.46E-03 |
| *SEL1L3* | 3.88 | 8.38E-07 | 1.92E-04 |
| *TM4SF1* | 3.86 | 2.29E-04 | 5.62E-03 |
| *PRR7* | 3.81 | 1.71E-06 | 2.81E-04 |
| *NPDC1* | 3.79 | 8.80E-11 | 7.52E-07 |
| *HOXA5* | 3.78 | 1.47E-07 | 6.62E-05 |
| *HOXA2* | 3.72 | 1.29E-05 | 1.02E-03 |
| *SHROOM4* | 3.63 | 1.65E-09 | 5.58E-06 |
| *TMEM163* | 3.60 | 9.35E-10 | 5.33E-06 |
| *MYCT1* | 3.59 | 1.96E-09 | 5.58E-06 |
| *HOXA3* | 3.55 | 4.75E-08 | 3.53E-05 |
| *PCCA* | 3.53 | 3.46E-05 | 1.73E-03 |
| *ACP6* | 3.51 | 8.85E-05 | 3.01E-03 |

HR, hazard ratio for highly expressed group; FDR, false discovery rate adjusted using the Benjamini–Hochberg method across all genes tested in the genome-wide survival analysis.

**Supplementary Table 8. Top 20 genes in order of Hazard Ratio for patients who did not receive 7+3 therapy in SIH/JIH/ZIH AML dataset**

| **Genes** | **HR** | ***P*** | **FDR** |
| --- | --- | --- | --- |
| *PPP2R5D* | 5.14 | 8.33E-05 | 1.32E-03 |
| *RPN1* | 4.95 | 1.39E-14 | 2.41E-10 |
| *PHGDH* | 4.70 | 8.83E-13 | 6.26E-09 |
| *UBE2L3* | 4.57 | 1.08E-12 | 6.26E-09 |
| *RNF187* | 4.45 | 1.22E-04 | 1.71E-03 |
| *PSMB3* | 4.43 | 5.48E-12 | 2.38E-08 |
| *SAP30* | 4.38 | 1.75E-11 | 3.82E-08 |
| *GALNT2* | 4.35 | 1.89E-05 | 4.99E-04 |
| *PTPRA* | 4.35 | 2.09E-11 | 3.82E-08 |
| *RPA1* | 4.31 | 1.85E-04 | 2.22E-03 |
| *LDHA* | 4.30 | 9.27E-12 | 2.68E-08 |
| *STRADB* | 4.25 | 1.22E-04 | 1.71E-03 |
| *CCT5* | 4.23 | 7.18E-11 | 9.30E-08 |
| *ICMT* | 4.18 | 7.44E-12 | 2.59E-08 |
| *PPP1R7* | 4.17 | 1.80E-11 | 3.82E-08 |
| *TMED2* | 4.15 | 2.42E-11 | 3.82E-08 |
| *FKBP5* | 4.13 | 2.41E-11 | 3.82E-08 |
| *PRKDC* | 4.05 | 1.86E-10 | 1.74E-07 |
| *EIF4EBP2* | 4.05 | 7.41E-11 | 9.30E-08 |
| *RTN3* | 4.03 | 8.06E-11 | 9.33E-08 |

HR, hazard ratio for highly expressed group; FDR, false discovery rate, adjusted using the Benjamini–Hochberg (BH) method across all genes tested in the genome-wide survival analysis.

**Supplementary Table 9. 20 genes with a high HR specifically related to the response to 7+3 therapy in** **SIH/JIH/ZIH AML dataset**

| **Genes** | **HR** | ***P*** | **FDR** |
| --- | --- | --- | --- |
| *PVR* | 4.43 | 1.44E-06 | 2.59E-04 |
| *ARHGEF16* | 4.29 | 1.87E-05 | 1.27E-03 |
| *CLU* | 4.24 | 2.61E-09 | 5.58E-06 |
| *CEACAM19* | 4.08 | 1.05E-06 | 2.21E-04 |
| *PRR7* | 3.81 | 1.71E-06 | 2.81E-04 |
| *SHROOM4* | 3.63 | 1.65E-09 | 5.58E-06 |
| *KAZALD1* | 3.41 | 2.40E-09 | 5.58E-06 |
| *GNAZ* | 3.4 | 9.83E-09 | 1.53E-05 |
| *P3H3* | 3.34 | 5.35E-09 | 1.02E-05 |
| *TMEM231* | 3.22 | 1.10E-07 | 5.78E-05 |
| *DSC2* | 3.22 | 9.53E-05 | 3.15E-03 |
| *ADGRG1* | 3.2 | 2.08E-08 | 2.19E-05 |
| *RUSC2* | 3.14 | 1.95E-04 | 5.04E-03 |
| *SCN9A* | 3.04 | 1.88E-07 | 7.47E-05 |
| *CCDC106* | 3.02 | 3.66E-05 | 1.79E-03 |
| *TTLL7* | 3.01 | 1.47E-07 | 6.62E-05 |
| *STXBP1* | 3.01 | 2.13E-06 | 3.14E-04 |
| *CNN3* | 2.98 | 5.60E-05 | 2.28E-03 |
| *PPFIA3* | 2.98 | 2.20E-05 | 1.38E-03 |
| *WNT11* | 2.97 | 1.13E-07 | 5.78E-05 |

HR, hazard ratio for highly expressed group; FDR, false discovery rate, adjusted using the BH method across all genes tested in the genome-wide survival analysis.

**Supplementary Figure 2. Overall survival analysis of representative high-risk genes in 7+3-treated AML patients from SIH/JIH/ZIH dataset.** Kaplan-Meier survival curves for representative genes related to prognosis of patients with 7+3 regimen: PVR **(A)** and ARHGEF16 **(B)** with highest HR. P values were obtained through log-rank test. HR, hazard ratio for highly expressed group.

**Supplementary Table 10. Top 15 upregulated pathways by GSEA analysis in poor response patients from OHSU BeatAML2.0.**

| **Name of Pathway** | **NES** | ***P*** | **FDR** |
| --- | --- | --- | --- |
| REACTOME_FORMATION_OF_FIBRIN_CLOT_CLOTTING_CASCADE | 2.02 | 0.000 | 0.050 |
| REACTOME_ELASTIC_FIBRE_FORMATION | 1.98 | 0.000 | 0.059 |
| REACTOME_DCC_MEDIATED_ATTRACTIVE_SIGNALING | 1.94 | 0.000 | 0.073 |
| REACTOME_PLATELET_ACTIVATION_SIGNALING_AND_AGGREGATION | 1.91 | 0.000 | 0.099 |
| REACTOME_SIGNALING_BY_BMP | 1.91 | 0.000 | 0.080 |
| REACTOME_EXTRA_NUCLEAR_ESTROGEN_SIGNALING | 1.90 | 0.000 | 0.074 |
| REACTOME_PLATELET_AGGREGATION_PLUG_FORMATION | 1.88 | 0.002 | 0.090 |
| REACTOME_COMMON_PATHWAY_OF_FIBRIN_CLOT_FORMATION | 1.87 | 0.002 | 0.091 |
| REACTOME_L1CAM_INTERACTIONS | 1.84 | 0.000 | 0.117 |
| REACTOME_INTRINSIC_PATHWAY_OF_FIBRIN_CLOT_FORMATION | 1.82 | 0.004 | 0.143 |
| REACTOME_CELL_CELL_JUNCTION_ORGANIZATION | 1.81 | 0.002 | 0.145 |
| REACTOME_GLUTATHIONE_CONJUGATION | 1.81 | 0.002 | 0.133 |
| REACTOME_PLATELET_ADHESION_TO_EXPOSED_COLLAGEN | 1.81 | 0.000 | 0.132 |
| REACTOME_GLUCAGON_LIKE_PEPTIDE_1_GLP1_REGULATES_INSULIN_SECRETION | 1.79 | 0.000 | 0.143 |
| REACTOME_RHO_GTPASES_ACTIVATE_PAKS | 1.79 | 0.007 | 0.135 |

NES, normalization of enrichment score; FDR, false discovery rate

**Supplementary Table 11. Top 15 upregulated pathways by GSEA analysis in poor response patients from SIH/JIH/ZIH AML dataset**

| **Name of Pathway** | **NES** | ***P*** | **FDR** |
| --- | --- | --- | --- |
| REACTOME_EXTRACELLULAR_MATRIX_ORGANIZATION | 2.72 | 0.000 | 0.000 |
| REACTOME_ELASTIC_FIBRE_FORMATION | 2.55 | 0.000 | 0.002 |
| REACTOME_FORMATION_OF_FIBRIN_CLOT_CLOTTING_CASCADE | 2.38 | 0.000 | 0.010 |
| REACTOME_GAP_JUNCTION_TRAFFICKING_AND_REGULATION | 2.27 | 0.000 | 0.028 |
| REACTOME_ACTIVATION_OF_MATRIX_METALLOPROTEINASES | 2.25 | 0.000 | 0.025 |
| REACTOME_RECYCLING_PATHWAY_OF_L1 | 2.23 | 0.002 | 0.029 |
| REACTOME_DEGRADATION_OF_THE_EXTRACELLULAR_MATRIX | 2.23 | 0.000 | 0.025 |
| REACTOME_RECEPTOR_TYPE_TYROSINE_PROTEIN_PHOSPHATASES | 2.21 | 0.000 | 0.028 |
| REACTOME_MOLECULES_ASSOCIATED_WITH_ELASTIC_FIBRES | 2.18 | 0.004 | 0.033 |
| REACTOME_COLLAGEN_FORMATION | 2.16 | 0.000 | 0.036 |
| REACTOME_PROTEIN_PROTEIN_INTERACTIONS_AT_SYNAPSES | 2.14 | 0.002 | 0.041 |
| REACTOME_DEFECTIVE_EXT2_CAUSES_EXOSTOSES_2 | 2.11 | 0.002 | 0.049 |
| REACTOME_ERYTHROCYTES_TAKE_UP_CARBON_DIOXIDE_AND_RELEASE_OXYGEN | 2.11 | 0.002 | 0.047 |
| REACTOME_COLLAGEN_DEGRADATION | 2.10 | 0.000 | 0.048 |
| REACTOME_HS_GAG_DEGRADATION | 2.09 | 0.002 | 0.047 |

NES, normalization of enrichment score; FDR, false discovery rate

**Supplementary Table 12. Top 15 downregulated pathways by GSEA analysis in poor response patients from OHSU BeatAML2.0.**

| **Name of Pathway** | **NES** | ***P*** | **FDR** |
| --- | --- | --- | --- |
| REACTOME_FCGR_ACTIVATION | -2.40 | 0.000 | 0.000 |
| REACTOME_INITIAL_TRIGGERING_OF_COMPLEMENT | -2.39 | 0.000 | 0.000 |
| REACTOME_CREATION_OF_C4_AND_C2_ACTIVATORS | -2.37 | 0.000 | 0.000 |
| REACTOME_CD22_MEDIATED_BCR_REGULATION | -2.34 | 0.000 | 0.000 |
| REACTOME_ANTIGEN_ACTIVATES_B_CELL_RECEPTOR_BCR_LEADING_TO_GENERATION_OF_SECOND_MESSENGERS | -2.26 | 0.000 | 0.000 |
| REACTOME_ROLE_OF_PHOSPHOLIPIDS_IN_PHAGOCYTOSIS | -2.26 | 0.000 | 0.000 |
| REACTOME_FCERI_MEDIATED_MAPK_ACTIVATION | -2.25 | 0.000 | 0.000 |
| REACTOME_ROLE_OF_LAT2_NTAL_LAB_ON_CALCIUM_MOBILIZATION | -2.25 | 0.000 | 0.000 |
| REACTOME_FCGR3A_MEDIATED_IL10_SYNTHESIS | -2.22 | 0.000 | 0.000 |
| REACTOME_SCAVENGING_OF_HEME_FROM_PLASMA | -2.15 | 0.000 | 0.000 |
| REACTOME_FCERI_MEDIATED_CA_2_MOBILIZATION | -2.15 | 0.000 | 0.000 |
| REACTOME_COMPLEMENT_CASCADE | -2.11 | 0.000 | 0.000 |
| REACTOME_FCERI_MEDIATED_NF_KB_ACTIVATION | -2.01 | 0.000 | 0.001 |
| REACTOME_ACTIVATION_OF_ANTERIOR_HOX_GENES_IN_HINDBRAIN_DEVELOPMENT_DURING_EARLY_EMBRYOGENESIS | -1.99 | 0.000 | 0.001 |
| REACTOME_SIGNALING_BY_THE_B_CELL_RECEPTOR_BCR | -1.96 | 0.000 | 0.002 |

NES, normalization of enrichment score; FDR, false discovery rate

**Supplementary Table 13. Top 15 downregulated pathways by GSEA analysis in poor response patients from SIH/JIH/ZIH AML dataset**

| **Name of Pathway** | **NES** | ***P*** | **FDR** |
| --- | --- | --- | --- |
| REACTOME_INTERLEUKIN_20_FAMILY_SIGNALING | -2.31 | 0.000 | 0.064 |
| REACTOME_GENERATION_OF_SECOND_MESSENGER_MOLECULES | -2.29 | 0.000 | 0.041 |
| REACTOME_GLYOXYLATE_METABOLISM_AND_GLYCINE_DEGRADATION | -2.19 | 0.000 | 0.072 |
| REACTOME_SIGNALING_BY_CYTOSOLIC_FGFR1_FUSION_MUTANTS | -2.16 | 0.004 | 0.069 |
| REACTOME_PD_1_SIGNALING | -2.14 | 0.000 | 0.068 |
| REACTOME_CLASS_C_3_METABOTROPIC_GLUTAMATE_PHEROMONE_RECEPTORS | -2.00 | 0.004 | 0.171 |
| REACTOME_INTERLEUKIN_2_SIGNALING | -1.88 | 0.007 | 0.359 |
| REACTOME_FGFR1_MUTANT_RECEPTOR_ACTIVATION | -1.87 | 0.011 | 0.337 |
| REACTOME_ELEVATION_OF_CYTOSOLIC_CA2_LEVELS | -1.87 | 0.002 | 0.304 |
| REACTOME_COHESIN_LOADING_ONTO_CHROMATIN | -1.85 | 0.006 | 0.297 |
| REACTOME_ASPIRIN_ADME | -1.81 | 0.011 | 0.359 |
| REACTOME_SYNTHESIS_OF_PIPS_AT_THE_LATE_ENDOSOME_MEMBRANE | -1.81 | 0.009 | 0.338 |
| REACTOME_DISEASES_OF_DNA_REPAIR | -1.77 | 0.002 | 0.396 |
| REACTOME_INTERLEUKIN_2_FAMILY_SIGNALING | -1.77 | 0.004 | 0.377 |
| REACTOME_HYALURONAN_METABOLISM | -1.76 | 0.022 | 0.360 |

NES, normalization of enrichment score; FDR, false discovery rate

**Supplementary Figure 3. Stepwise analytical workflow for candidate gene selection.** The numbers of genes retained at each step and the corresponding selection criteria are shown.

**Supplementary Figure 4. Overall survival analysis based on the expression of 7 gene signatures in SIH/JIH/ZIH AML dataset. A-G** P values were determined by the log-rank test. HR, hazard ratio of highly expressed group.

**Supplementary Figure 5. Cross-cohort correlations of *F2R* and *TMEM150C* with core genes from elastic fibre formation and fibrin clotting pathways.** Spearman correlations were calculated between *F2R* or *TMEM150C* expression and GSEA core genes from the Reactome Elastic Fibre Formation and Formation of Fibrin Clot pathways in the SIH/JIH/ZIH AML and OHSU BeatAML2.0 cohorts. **A** Core genes were classified according to the significance and direction of their correlations with *F2R* or *TMEM150C* across the two cohorts. **B** Heatmaps show gene-level Spearman correlations in each cohort. BH correction for multiple testing was applied separately within each cohort–candidate gene–pathway combination. Color indicates Spearman’s rho, and asterisks indicate BH-adjusted FDR < 0.05.

**Supplementary Table 14. Literature summary of seven candidate genes identified in 7+3 therapy resistance**

| **No** | **Expression** | **Gene** | **Refractory response/ Poor survival in AML** | **Biological functions in other tumor** | **Reference** |
| --- | --- | --- | --- | --- | --- |
| 1 | Up | *TMEM150C* (Transmembrane Protein 150C) | No association has been reported in AML | Modulates mechanogated channels; TMEM family is implicated in cancer migration and epithelial–mesenchymal transition (EMT) | ^1,2^ |
| 2 |  | *F2R* (Coagulation Factor II Receptor) | Reported to play a contradictory role in AML; promotes LSC growth and survival, and leukemia initiation when LSCs are scarce | Activates coagulation-related signaling and inflammation; contributes to tumor invasion and angiogenesis | ^3-5^ |
| 3 |  | *PLCB4* (Phospholipase C Beta 4) | Upregulation is associated with poor prognosis in pediatric AML; highly expressed in multidrug-resistant leukemia cells | Involved in phospholipid signaling, promoting cancer migration and drug resistance; high expression correlates with poor survival and reduced immune infiltration in osteosarcoma | ^6,7^ |
| 4 |  | *CPS1* (Carbamoyl Phosphate Synthetase 1) | No association has been reported in AML | Catalyzes the conversion of ammonium into carbamoyl phosphate as the first rate-limiting enzyme of the urea cycle; high expression is associated with poor prognosis in NSCLC and lung cancer by sustaining the pyrimidine pool | ^8-10^ |
| 5 |  | *GIPC2* (GIPC PDZ Domain Containing 2) | Functions as a tumor suppressor in AML; promoter hypermethylation reduces GIPC2 expression, while restoration of expression suppresses leukemia cell survival by inhibiting the PI3K/AKT pathway | Promotes metastasis in prostate cancer through WNT signaling activation | ^11,12^ |
| 6 | Down | *ZBTB16* (Zinc Finger and BTB Domain Containing 16) | In acute promyelocytic leukemia (APL), ZBTB16-RARA fusion causes differentiation arrest, treatment resistance, and disseminated intravascular coagulation | Acts as a tumor suppressor regulating cell cycle and apoptosis in lung adenocarcinoma | ^13-15^ |
| 7 |  | *ESPNL* (espin like) | Promoter region reported to be hypomethylated in AML and in MDS (myelodysplastic syndromes) | No functional evidence reported | ^16^ |

**References**

1.	Anderson EO, Schneider ER, Matson JD, Gracheva EO, Bagriantsev SN. TMEM150C/Tentonin3 Is a Regulator of Mechano-gated Ion Channels. *Cell reports*. 2018;23(3):701-708. doi: 10.1016/j.celrep.2018.03.094

2.	Herrera-Quiterio GA, Encarnacion-Guevara S. The transmembrane proteins (TMEM) and their role in cell proliferation, migration, invasion, and epithelial-mesenchymal transition in cancer. *Frontiers in oncology*. 2023;13:1244740. doi: 10.3389/fonc.2023.1244740

3.	Goyama S, Shrestha M, Schibler J, Rosenfeldt L, Miller W, O'Brien E, et al. Protease-activated receptor-1 inhibits proliferation but enhances leukemia stem cell activity in acute myeloid leukemia. *Oncogene*. 2017;36(18):2589-2598. doi: 10.1038/onc.2016.416

4.	Wu X, Wang S, Wang C, Wu C, Zhao Z. Bioinformatics analysis identifies coagulation factor II receptor as a potential biomarker in stomach adenocarcinoma. *Sci Rep*. 2024;14(1):2468. doi: 10.1038/s41598-024-52397-6

5.	Dong YM, Bao GQ. Coagulation factor II thrombin receptor as a promising biomarker in breast cancer management. *Open life sciences*. 2024;19(1):20221001. doi: 10.1515/biol-2022-1001

6.	Wu S, Zhang W, Shen D, Lu J, Zhao L. PLCB4 upregulation is associated with unfavorable prognosis in pediatric acute myeloid leukemia. *Oncology letters*. 2019;18(6):6057-6065. doi: 10.3892/ol.2019.10921

7.	Liu G, Fang Y, Li J, Chen Z. Anoikis-related PLCB4 is linked to immunotherapy response in osteosarcoma. *Discov Oncol*. 2025;16(1):226. doi: 10.1007/s12672-025-01970-0

8.	Celiktas M, Tanaka I, Tripathi SC, Fahrmann JF, Aguilar-Bonavides C, Villalobos P, et al. Role of CPS1 in Cell Growth, Metabolism and Prognosis in LKB1-Inactivated Lung Adenocarcinoma. *Journal of the National Cancer Institute*. 2017;109(3):1-9. doi: 10.1093/jnci/djw231

9.	Kim J, Hu Z, Cai L, Li K, Choi E, Faubert B, et al. CPS1 maintains pyrimidine pools and DNA synthesis in KRAS/LKB1-mutant lung cancer cells. *Nature*. 2017;546(7656):168-172. doi: 10.1038/nature22359

10.	Hajaj E, Sciacovelli M, Frezza C, Erez A. The context-specific roles of urea cycle enzymes in tumorigenesis. *Molecular cell*. 2021;81(18):3749-3759. doi: 10.1016/j.molcel.2021.08.005

11.	Wang L, Wang J, Yin X, Guan X, Li Y, Xin C, et al. GIPC2 interacts with Fzd7 to promote prostate cancer metastasis by activating WNT signaling. *Oncogene*. 2022;41(18):2609-2623. doi: 10.1038/s41388-022-02255-4

12.	Zhao J, Liu J, Liu S, Tian S, Yuan H, Li S. GIPC2 is a tumor suppressor gene for acute myeloid leukemia and induces apoptosis of leukemia cells by regulating the PI3K/AKT pathway. *Epigenomics*. 2023;15(6):369-383. doi: 10.2217/epi-2023-0027

13.	Langabeer SE, Preston L, Kelly J, Goodyer M, Elhassadi E, Hayat A. Molecular Profiling: A Case of ZBTB16-RARA Acute Promyelocytic Leukemia. *Case reports in hematology*. 2017;2017:7657393. doi: 10.1155/2017/7657393

14.	Fabiani E, Cicconi L, Nardozza AM, Cristiano A, Rossi M, Ottone T, et al. Mutational profile of ZBTB16-RARA-positive acute myeloid leukemia. *Cancer medicine*. 2021;10(12):3839-3847. doi: 10.1002/cam4.3904

15.	Wang K, Guo D, Yan T, Sun S, Wang Y, Zheng H, et al. ZBTB16 inhibits DNA replication and induces cell cycle arrest by targeting WDHD1 transcription in lung adenocarcinoma. *Oncogene*. 2024;43(23):1796-1810. doi: 10.1038/s41388-024-03041-0

16.	Cecotka A, Polanska J. Region-Specific Methylation Profiling in Acute Myeloid Leukemia. *Interdisciplinary sciences*. 2018;10(1):33-42. doi: 10.1007/s12539-018-0285-4
